# Incremental cost-effectiveness of screening and laser treatment for diabetic retinopathy and macular edema in Malawi

**DOI:** 10.1371/journal.pone.0190742

**Published:** 2018-01-04

**Authors:** Damir Vetrini, Christine A. Kiire, Philip I. Burgess, Simon P. Harding, Petros C. Kayange, Khumbo Kalua, Gerald Msukwa, Nicholas A. V. Beare, Jason Madan

**Affiliations:** 1 SDA Bocconi School of Management, Bocconi University, Milan, Italy; 2 Oxford Eye Hospital, John Radcliffe Hospital, Oxford University Hospitals NHS Foundation Trust, Oxford, United Kingdom; 3 Department of Eye and Vision Science, Institute of Ageing and Chronic Disease, University of Liverpool, Liverpool, United Kingdom; 4 Malawi Liverpool Wellcome Trust Clinical Research Programme, Queen Elizabeth Central Hospital, Blantyre, Malawi; 5 St. Paul’s Eye Unit, Royal Liverpool University Hospital, Liverpool, United Kingdom; 6 College of Medicine, University of Malawi, Blantyre, Malawi; 7 Lions Sight First Eye Hospital, Blantyre, Malawi; 8 Centre for Applied Health Research and Delivery, Division of Health Sciences, Warwick Medical School, Coventry, United Kingdom; University of Michigan, UNITED STATES

## Abstract

**Objective:**

To investigate the economic impact of introducing targeted screening and laser photocoagulation treatment for sight-threatening diabetic retinopathy and macular edema in a setting with no previous screening or laser treatment for diabetic retinopathy in sub-Saharan Africa.

**Materials and methods:**

A cohort Markov model was built to compare combined targeted screening and laser treatment for patients with sight-threatening diabetic retinopathy and macular edema against no intervention. Primary outcomes were incremental cost per quality-adjusted life year (QALY) gained and per disability-adjusted life year (DALY) averted. Primary data were collected on 357 participants from the Malawi Diabetic Retinopathy Study, a prospective, observational cohort study. Multiple scenarios were explored and a probabilistic sensitivity analysis was performed.

**Results:**

In the base case (age: 50 years, service utilization rate: 80%), the cost of the intervention and the years of severe visual impairment averted per patient screened were $209 and 2.2 years respectively. Applying the World Health Organization threshold of cost-effectiveness for Malawi ($679), the base case was cost-effective when QALYs were used ($400 per QALY gained) but not when DALYs were used ($766 per DALY averted). The intervention was more cost-effective when it targeted younger patients (age: 30 years) and less cost-effective when the utilization rate was lowered to 50%.

**Conclusions:**

Annual photographic screening of diabetic patients attending medical diabetes clinics in Malawi, with the provision of laser treatment for those with sight-threatening diabetic retinopathy and macular edema, appears to be cost-effective in terms of QALYs gained, in our base case scenario. Cost-effectiveness improves if services are utilized more intensively and extended to younger patients.

## Introduction

Non-communicable diseases have a major impact on the global burden of disease, contributing 54% of all disability-adjusted life years (DALYs) lost globally [1]. Diabetes is a leading cause of morbidity and mortality in most high-income countries and its prevalence is rising rapidly in economically developing countries [2]. It causes visual impairment primarily through the development of diabetic retinopathy (DR), in particular, proliferative diabetic retinopathy (PDR) and diabetic maculopathy, which includes both macular edema and macular ischemia. DR is the fifth leading cause of global blindness [3]. Medical interventions can decrease some of the risk to vision [4–7]. If sight-threatening retinopathy (STDR) is present, timely laser photocoagulation of the retina decreases the risk of severe vision loss [8,9].

The International Diabetes Federation has estimated that the number of adults with diabetes in Africa will increase by 98%, from 12.1 million in 2010 to 23.9 million in 2030 [10]. Malawi is one of the poorest countries in the world, with an annual per capita healthcare expenditure of international $93 in 2014 [11]. The recent World Health Organization (WHO) Malawi national STEPwise survey estimated a prevalence of diabetes of 5.6% in adults aged 25 to 64 years, with similar prevalence levels in rural and urban areas [12].

Economic analyses demonstrate that screening for, and treatment of, STDR are cost-effective in developed countries [13–15]. However, to the best of our knowledge, the cost-effectiveness of a combined screening and treatment program for STDR in sub-Saharan Africa has not been previously described.

The Malawi Diabetic Retinopathy Study (MDRS) is an epidemiological study of DR and its treatment in Malawi. Our objective in this analysis was to use data from the MDRS to investigate the cost-effectiveness of screening and laser treatment for STDR targeted to patients attending hospital-based diabetes clinics, compared to no systematic screening and no treatment, which is current practice in Malawi.

## Materials and methods

### Study setting and intervention

The MDRS is a prospective, observational cohort study of DR in patients attending primary/secondary care diabetes clinics in the urban and semi-urban southern Malawi locations of Queen Elizabeth Central Hospital, Blantyre, and Zomba Central Hospital, Zomba. It adhered to the tenets of the declaration of Helsinki. Ethical approval for the study was granted by the College of Medicine Research Ethics Committee in Malawi and the University of Liverpool Committee on Research Ethics. Three hundred and fifty-seven participants, with both type 1 and type 2 diabetes, were identified by systematic random sampling of patients attending these clinics between December 2011 and May 2012. Written informed consent was obtained from all subjects and they were followed up in the eye clinic for two years. Demographic and clinical characteristics of participants in the MDRS have been previously described [16].

Study participants underwent an annual assessment of their eyes on the day they attended their medical diabetic clinic including visual acuity measured by a clinical assistant, and slit-lamp biomicroscopy and digital fundus photography which were performed by an experienced ophthalmologist. Those who required retinal laser photocoagulation typically had up to three additional clinic visits over a 12-month period. Retinal photographs were graded at the accredited reading center in the University of Liverpool, UK for the purposes of quality assurance of the study. Retinopathy and maculopathy were classified by feature-specific grading using definitions established in the Liverpool Diabetic Eye Study (LDES) [17].

The threshold for scatter laser treatment (peripheral retinal photocoagulation, PRP) was the ETDRS ‘4-2-1 rule’, equivalent to level 50 retinopathy on the LDES scale. Standard treatment was approximately 3000 laser burns placed one burn width apart, outside the vascular arcades and at least 2 disc diameters from the center of the fovea. Scatter laser treatment was typically delivered over two or more hospital visits. Eyes with clinically significant macular edema (CSME) and visual acuity < 80 ETDRS letters (equivalent to 20/30 Snellen), or exudates tracking towards the center of the fovea, underwent focal laser or modified macular grid laser as appropriate [18]. All MDRS participants who fulfilled the criteria for laser treatment were treated, and rates of progression and/or regression of DR, for these and the participants who did not require treatment, were reviewed on an annual basis.

### Cost-effectiveness analysis

A cohort Markov model, built in Microsoft Excel 2011, was used to estimate the cost-effectiveness of combined targeted screening and laser treatment for STDR in the MDRS patient population. In this model photographic screening, grading of retinal photographs and laser treatment is performed by an ophthalmologist. The decision whether to instigate laser treatment is made at the appointment at which the screening procedure is performed. Treatment is undertaken at a separate appointment. The size of the hypothetical cohort was 1000 diabetic patients entering the model at the age of 50 years and each model cycle was one year. Costs and outcomes were estimated over a 25-year period based on the age-specific life expectancy in Malawi [19]. An annual discount rate of 3% was applied to both costs and health effects in line with the WHO cost-effectiveness analysis (CEA) guidelines [20].

The primary outcome of this study was the incremental cost-effectiveness ratio (ICER), in terms of cost per quality-adjusted life years (QALYs) gained and per DALY averted, of the combination of a targeted screening program and laser treatment for STDR. Secondary outcomes included the cost of service delivery and the number of years of severe visual impairment avoided through screening and laser treatment.

For the purpose of this analysis, LDES level 10 was classified as no retinopathy, levels 20 and 30 as mild non-proliferative diabetic retinopathy (NPDR), level 40 as moderate NPDR, and level 50+ as severe non-proliferative diabetic retinopathy (SNPDR) / PDR. Level 4 maculopathy on the LDES scale was considered to be equivalent to CSME as defined in the Early Treatment of Diabetic Retinopathy Study (ETDRS) [21]. Only data from the eye with poorer vision were included in this analysis.

**Fig 1:**
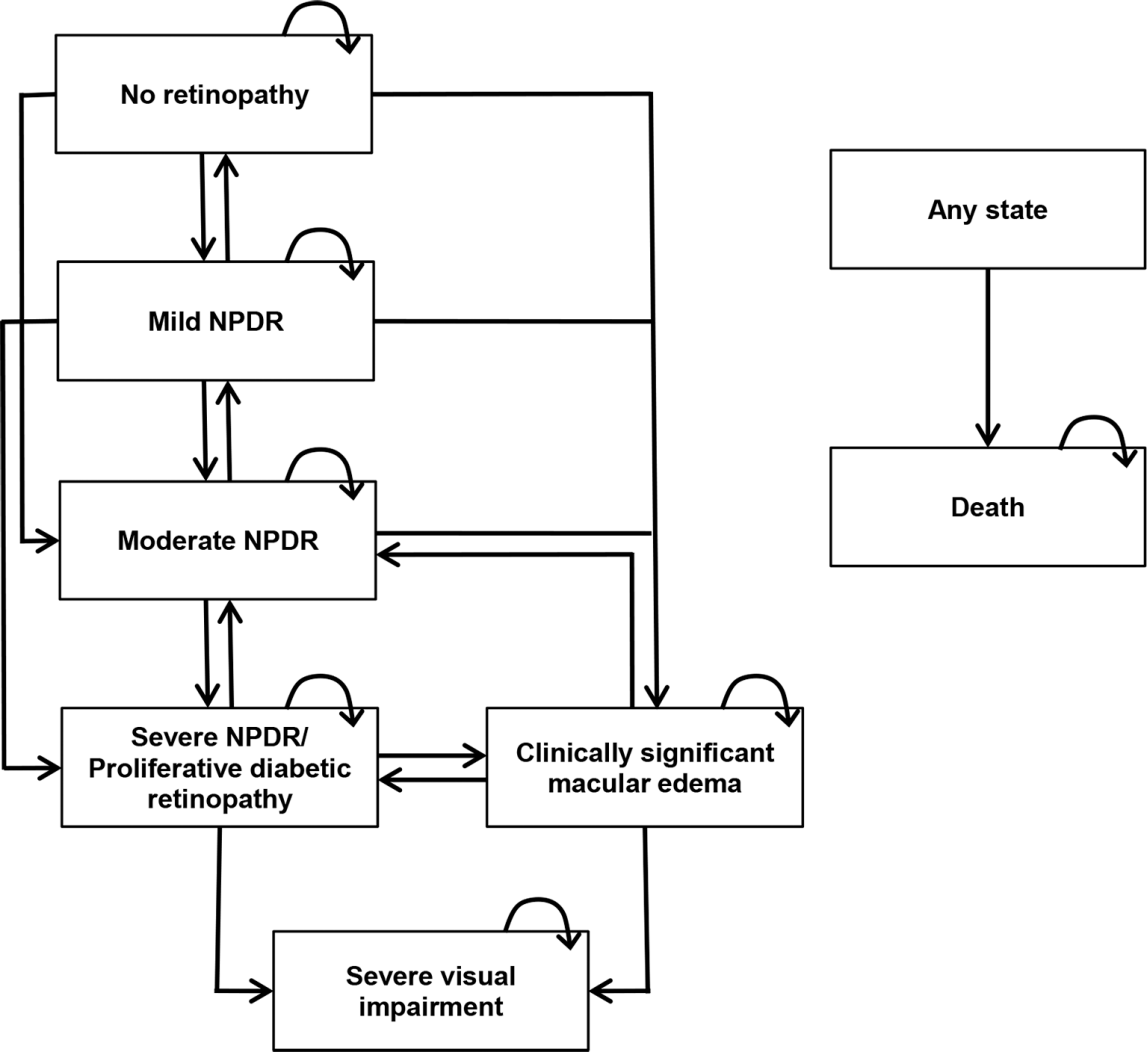
Model schematic. The arrows indicate the permitted movement between the health states. NPDR = non-proliferative diabetic retinopathy.

Simulated patients were allocated into one of six Markov health states reflecting the different disease stages: no DR, mild NPDR, moderate NPDR, SNPDR/PDR, CSME, severe visual impairment. SNPDR and PDR were grouped into the same state, as they were the treatment threshold for scatter PRP. Severe visual impairment was defined as best corrected visual acuity (VA) < 6/60 in the better eye (WHO [22]). The model structure is outlined in Fig 1.

Primary data were collected from the MDRS on the epidemiology of DR and macular edema in an urban and semi-urban setting in Malawi, laser photocoagulation effectiveness and costs of screening and laser treatment program. Other model parameters were derived from the published literature (specified below) [8,13,14,23,24,25]. The main model parameters are outlined in Table 1.

**Table 1:** Markov model clinical parameter estimates.

Parameter	Base case	95% CI	Source
**Annual transition probabilities**			
No DR to mild NPDR	0.21	0.16–0.25	MDRS
Mild NPDR to moderate NPDR	0.06	0.03–0.09	MDRS
Moderate NPDDR to severe NPDR/PDR	0.11	0.02–0.25	MDRS
Severe NPDR/PDR to severe visual impairment without treatment	0.09	0.07–0.12	[8,13,14]
Severe NPDR/PDR to severe visual impairment with treatment	0.02	0–0.07	MDRS
CSME to severe visual impairment without treatment	0.05	0.03–0.08	[8,13,14]
CSME to severe visual impairment with treatment	0.03	0–0.09	MDRS
**Mortality**			
Diabetes-specific relative risk	1.97	1.17–2.88	[24]
**Initial distribution**			
No DR	44%	41%-48%	MDRS
Mild NPDR	30%	27%-33%	MDRS
Moderate NPDR	9%	7%-11%	MDRS
Severe NPDR/PDR	4%	2%-5%	MDRS
CSME	13%	11%-15%	MDRS
**Health-state utilities**			
No DR	0.89	0.78–0.96	[23]
Mild NPDR	0.80	0.73–0.86	[23]
Moderate NPDR	0.80	0.73–0.86	[23]
Severe NPDR/PDR	0.70	0.63–0.76	[23]
CSME	0.70	0.63–0.76	[23]
Severe visual impairment (VA<6/60)	0.55	0.46–0.63	[23]
**Disability weight**			
Severe visual impairment (VA<6/60)	0.19	0.13–0.27	[25]

CI = Confidence interval; DR = Diabetic retinopathy; NPDR = Non-proliferative diabetic retinopathy; CSME = Clinically significant macular edema; VA = Visual acuity

Rates of progression/regression of disease from no retinopathy, mild retinopathy and moderate retinopathy were determined from the MDRS. Progression to severe visual impairment after treatment was derived from the MDRS based on the proportion of patients who developed a reduction in vision to VA < 6/60 within a year. Data on progression of disease from SNPDR/PDR and CSME to severe visual impairment were derived from the ETDRS [8,13,14].

Age specific mortality rates were obtained from the WHO Malawi life tables for the general population [19]. In the absence of Malawi-specific data, a diabetes-specific relative risk of death was calculated from data from a study conducted in Tanzania [24]. WHO 5-year age group mortality rates were multiplied by the mortality multiplier and then converted into 1-year mortality probabilities.

### Cost parameters

A healthcare provider perspective was chosen and all direct costs accrued by the hospital were included (Table 2).

**Table 2:** Per-patient hospital costs for a screening visit and a laser treatment visit.

	Screening (US$)	Treatment (US$)
Staff salaries	2.77	2.14
Buildings	0.17	0.77
Equipment	0.09	10.76
Consumables	0.72	0.48
Total	3.75	14.16

We used an ingredient costing approach, collecting information on the quantity and value of each resource used for the provision of the targeted screening program and laser treatment, where indicated, in the MDRS. Costs were recorded in Malawian Kwacha and converted into US dollars (average World Bank exchange rate in 2013 USD:MKw = 1:364.4). The costs of all the inputs were considered whether or not they represented a direct financial cost to the provider.

Costs were divided into four main categories: staff, buildings, equipment and consumables. They were sub-categorized into screening and laser visits. The costs of a screening visit were assumed to be the same as those of a non-laser follow up visit. Staff costs were calculated from annual gross salaries and the time taken for each type of hospital visit. They included the salary of the ophthalmologist and one clinical assistant. Building costs included rent, utilities and cleaning costs. The proportion of the total building costs allocated to the screening and laser treatment rooms was based on their floor area. The working life of the equipment was anticipated to be 10 years for the slit-lamp, fundus camera and laser, and 5 years for the lenses needed for slit-lamp examination and for laser treatment. The costs of consumables were based on the quantity of eye drops used for each visit. Unit costs were taken from official price guides recommended by the WHO, which provide international average prices.

### Effectiveness parameters

In order to determine the ICER for the screening and laser treatment program, we calculated QALYs gained, DALYs averted and years of severe visual impairment avoided. QALYs were calculated using utilities estimated by S. Polack et al. [23] in a study conducted in India, due to the lack of utility values specific for Malawi. For the calculation of DALYs, we used the disability weights provided by the WHO in the Global Burden of Diseases project (GBD) [25]. As the severity of DR does not always correlate with a specific degree of vision loss, it was not possible to use the disability weights reported by the GBD for mild and moderate visual impairment. For our assessment of severe visual impairment, however, we were able to use the GBD disability weight for severe visual impairment (0.191; 95% CI: 0.129–0.269).

### Other model inputs and assumptions

The baseline distribution of the population among the different health states was estimated from the MDRS patients aged between 45 and 55 years. The average number of eyes treated per patient in a single laser session in the MDRS was 1.6. The proportion of patients diagnosed with CSME who also received treatment for DR was 62%. We made the following assumptions for the model:

- patients in no retinopathy, mild NPDR and moderate NPDR health states received one screening visit per year
- patients in the SNPDR/PDR health state at their annual screening visit received three subsequent laser sessions and two follow-up visits within the year
- patients in the CSME health state at their annual screening visit received one laser session and two follow-up visits within the year
- patients in the severe visual impairment health state did not receive any further treatment and therefore no cost or improvement in health state was considered for these patients
- all patients meeting the inclusion criteria for treatment applied in the MDRS received laser scatter treatment and/or macular laser treatment.

### Sensitivity analysis

Five different intervention scenarios were evaluated in our cost-effectiveness analysis: base case, population age 30, +20% and -20% of salaries, and utilization rate set at 50%. In the base case, simulated patients were 50 years of age, the service utilization rate for the calculation of per-patient equipment costs (slit lamp and laser) was set at 80% [20] and the initial distribution of patients across the different health states was based on the retinopathy grade and vision data at baseline of MDRS participants aged between 45 and 55 years. Similarly, the age 30 scenario involved simulated patients who were 30 years of age and the distribution of patients across the different health states was based on the retinopathy and vision data from the MDRS participants aged between 25 and 35 years at the point of their enrolment into the study. The time horizon used for this scenario was 38 years, based on the age-specific life expectancy in Malawi [19]. The +/-20% salary and the 50% utilization rate scenarios were evaluated relative to the base case scenario. The +/-20% salary scenarios were included to explore the impact of potential variations in salaries on cost-effectiveness, which might be relevant if salaries differ between otherwise similar countries and contexts. The utilization rate was varied to investigate how the number of patients being screened and treated, if appropriate, affects the cost-effectiveness of this intervention.

For each scenario, we carried out a probabilistic sensitivity analysis (PSA) [26] to quantify decision uncertainty given the evidence available. Decision uncertainty was presented using cost-effectiveness acceptability curves (CEACs), a graphical representation of the probability that the intervention is cost-effective as a function of a decision-maker’s willingness-to-pay [27,28].

## Results

### Health outcomes and cost-effectiveness

The estimated costs, incremental QALYs, incremental DALYs, years of severe visual impairment avoided per diabetic patients, and ICERs for the intervention arm compared to standard care are shown in Table 3 for each scenario.

**Table 3:** Estimated costs, clinical outcomes, and ICERs per diabetic patient screened and treated with laser, if indicated, in each scenario.

Scenario	Cohort age (years)	Service utilization rate (%)	Salary (%)	Costs (US$)	Incremental QALYs	Incremental DALYs	Years of severe visual impairment avoided	ICER (per QALY)	ICER (per DALY)
Base case	50	80	100	209	0.523	0.273	2.199	$400	$766
Age 30	30	80	100	256	0.804	0.466	7.068	$318	$549
-20% salary	50	80	80	195	0.523	0.273	2.199	$372	$713
+20% salary	50	80	120	224	0.523	0.273	2.199	$428	$820
50% utilization rate	50	50	100	274	0.523	0.273	2.199	$523	$1,002

QALY = Quality-adjusted life year; DALY = Disability-adjusted life year; ICER = Incremental cost-effectiveness ratio

As expected, the highest clinical outcomes are obtained with the age 30 scenario (0.804 QALYs gained, 0.466 DALYs averted and 7.068 years of severe visual impairment avoided per patients) due to longer time spent in the model by the cohort. The other four scenarios are associated with the same outcomes as only costs were varied: 0.523 QALYs gained, 0.273 DALYs averted and 2.199 years of severe visual impairment avoided per patient. The per-patient costs of the intervention are: $195 for the -20% of salaries scenario, $209 for the base case, $224 for the +20% of salaries scenario, $256 for the age 30 scenario, and $274 for the 50% utilization rate scenario. From a health provider perspective and compared with no screening and no treatment, the intervention in the base case has an ICER of $400 per QALY gained. The lowest ICER is obtained with the age 30 scenario ($318 per QALY) while the 50% utilization rate scenario is the least cost-effective ($523 per QALY). The impact on the ICER of an increase ($428 per QALY) or a reduction ($372 per QALY) by 20% in the staff salaries is relatively small. Using the WHO threshold for low-income countries [20,29], in the base case and all the sensitivity scenarios explored the intervention appears cost-effective (<3x GDP/capita) but not highly cost-effective (<1x GDP/capita).

When DALYs are used, the ICERs become higher because the incremental DALYs averted are smaller. In the base case the ICER is $766 per DALY averted, $549 for the age 30 scenario, $713 and $820 respectively for the -/+ 20% of salaries scenarios and $1,002 for the 50% utilization rate scenario. In this case the only ICER falling within the WHO cost-effectiveness threshold calculated for Malawi is of the age 30 scenario.

### Probabilistic sensitivity analysis

**Fig 2:**
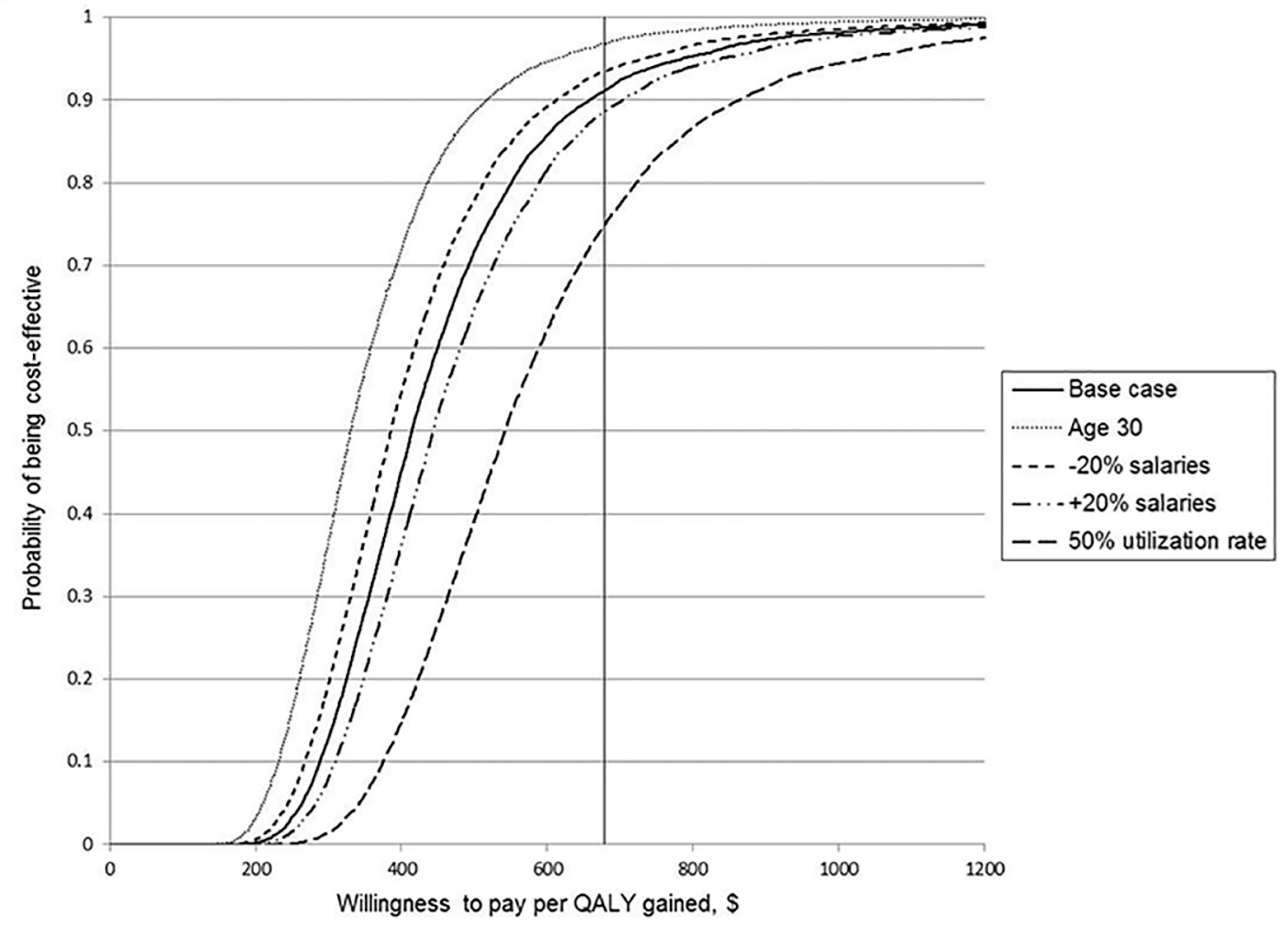
Cost-effectiveness acceptability curves based on QALYs. Probability that the intervention is cost-effective in a range of cost-effectiveness thresholds for the base case, + 20% on salaries scenario, -20% on salaries scenario, 50% utilization rate scenario, and age 30 scenario. QALY = Quality-adjusted life year.

The results of the probabilistic sensitivity analysis (PSA) based on QALYs are shown in Fig 2.

Considering the WHO cost-effectiveness threshold for an intervention in Malawi ($679 per QALY), the base case has a 91% probability of being cost-effective. The age 30 scenario has the highest probability of being cost effective (97%), and the 50% utilization rate scenario has the lowest probability of being cost-effective (75%), at this threshold.

When results of the PSA are based on DALYs, all the curves shift to the right, with a reduction in the probability of the intervention being cost-effective (S1 Fig). For the same WHO cost-effectiveness threshold of $679, the probability of the base case scenario being cost-effective would fall to 33%, compared with 66% for the age 30 scenario, and 10% for the 50% utilization rate scenario.

## Discussion

It has previously been shown that at baseline in the MDRS, the prevalence of STDR in the patients attending hospital based diabetic clinics was estimated to be 29.4%, approximately four times that reported in recent European studies [16]. Over a 24-month period, the incidence of STDR was 10.2%. This is approximately three times that seen in Europe [30]. Our objective in this analysis was to use the MDRS data to investigate the cost-effectiveness of combined targeted screening and laser treatment, where indicated, for STDR, compared to no systematic screening and no treatment for DR, in this context.

Our data suggest that providing this type of DR service in this setting in Malawi can be cost-effective, by the WHO criteria, in a range of service delivery scenarios, particularly when considering the ICER in terms of QALYs gained. They also suggest that in the base case, 2.2 years of severe visual loss per patient might be avoidable.

The PSA shows how sensitive the cost-effectiveness result is to the age profile of beneficiaries. In the age 30 scenario the incremental costs are amongst the highest but the increase in health gain for the model population generates a lower ICER. This reflects the increased number of years spent by patients in the model during which they benefit from the intervention. When DALYs averted are used to evaluate cost-effectiveness, the ICERs are higher because the incremental DALYs averted are smaller. In this case, the only ICER falling within the WHO cost-effectiveness threshold calculated for Malawi is for the age 30 scenario. The utilization rate also has a marked impact on the cost-effectiveness of the intervention. This is because the highest costs are the fixed costs of the equipment required to deliver this service (e.g. the laser) and these remain the same even when the equipment is used less frequently. Varying salaries by +/-20% has the smallest impact on the cost-effectiveness result.

These data clearly demonstrate that the probability of the intervention being cost-effective is affected by the health outcome measure selected for the analysis. Unlike QALYs, DALYs in our model can only capture the health impact of the worst health state. The findings of Polack et al. [23] suggest that patients place a value on avoiding less severe vision problems, which is only reflected in the QALYs calculation. While DALYs are commonly used in cost-effectiveness analyses [31], our study illustrates how their lack of sensitivity to certain types of health improvement might undervalue interventions that achieve these improvements.

### Strengths and limitations

There are few studies conducted in low- and middle-income countries with which to compare our results. To the best of our knowledge, the only cost-effectiveness analysis published to date for DR screening in sub-Saharan Africa is a study that was conducted by Khan and colleagues [32]. They showed that, when compared to no screening, non-mydriatic digital fundoscopy is a cost-effective measure for screening and diagnosis of DR in a primary care setting in South Africa. Like other recent studies in other parts of the world [14,33,34], their analyses focus the attention on the economic impact of screening programs and do not include laser treatment, as ours does. Another cost-effectiveness analysis of screening for DR in a resource-constrained setting is the analysis conducted by Rachapelle et al. [35] in India. They showed that a DR telescreening program is cost-effective compared to no screening in a rural Indian setting.

Participants in the MDRS were randomly sampled from two hospital-based clinics that were providing both primary and secondary care, and, as such, the distribution of the levels of retinopathy seen is likely to be typical of the population attending these clinics. It is possible, however, that the demographics and high levels of severity of disease in this population might not be completely representative of the general population with diabetes in Malawi.

Intravitreal anti vascular endothelial growth factor (anti-VEGF) agents are an important treatment for DR and macular edema in many countries. These agents are not available in the public sector in Malawi (and most countries in the region) in part due to cost. A separate, significant barrier to their introduction is the lack of suitable sterile compounding pharmacy facilities and appropriately trained pharmacists to prepare the drug safely. The use of anti-VEGF agents was therefore not considered in this analysis.

In the sub-Saharan African setting there is limited published data on costs and clinical outcomes available for this type of economic analyses. For our study we were able to obtain data on most of the key variables directly from the MDRS. By using these data we have been able to minimize bias associated with the use of cost data and retinopathy progression data (with treatment) from a different setting. It was not, however, possible to obtain all of the model inputs from Malawian or other sub-Saharan African sources. Our data on outcomes for untreated STDR came from the ETDRS, a well-known study of the treatment of DR that was conducted in the US over 35 years ago, a time before the use of laser treatment was widespread [8]. We chose to use data from the ETDRS because it provides robust outcomes data on untreated patients. No equivalent data exists in other settings, and it would now be unethical to not include treatment for patients in a study such as this today. Moreover, the progression rates to severe visual impairment in patients who received laser treatment in the MDRS and in the ETDRS are comparable. Therefore, we considered appropriate the use of data from the ETDRS to inform disease progression in the untreated population. In the absence of locally available data, a study based in India published by Polack et al. [23] was chosen as our source of utility values. The authors classified DR and macular edema according to the same health states that we used in our model and, as in our study, their analysis was based on the eye with worse vision. It is possible that the MDRS participants might perceive the disutility caused by the different levels of DR differently from those in the Indian study. However, we believe that data from an Indian setting (lower middle-income country) may be more relevant to a sub-Saharan African context than the data available from high-income countries.

A potential limitation of our analysis is the use of vision-related utility values that Polack et al. elicited through a direct method (time trade-off, TTO) in which the highest value anchors in perfect vision rather than perfect health. Their study also reported the EQ-5D utility values (in which the highest value anchors in perfect health) calculated using UK weights in the absence of Indian-specific tariffs. Kymes and Lee [36] discussed in their paper the potential bias of using vision-related utility values in cost-effectiveness analyses. However, the use of UK weights may not appropriately reflect patients’ quality of life in an Indian setting, therefore we used TTO-elicited utility values for our analysis. The mortality relative risk for diabetes came from a study conducted in neighboring Tanzania [24]. There are differences between persons with diabetes in Tanzania and Malawi, including in ethnic, cultural and economic diversity, as well as levels of access to health care. These could lead to differences in the relative risk between the two countries, but this was our best estimate for this variable in Malawi.

For the purpose of our analysis, we made the assumption that patients in the severe visual impairment health state did not receive any further treatment. This does not precisely reflect what happened in the MDRS. As a result of the low number of participants in this health state, there was an insufficient amount of data for us to model regression from severe visual impairment in response to laser treatment. For this reason, the severe visual impairment health state was considered to be an absorbing state and we believe this to be a conservative assumption.

This cost-effectiveness analysis was conducted from the perspective of healthcare providers. It did not include personal costs for patients attending the clinic, such as the cost of transport to the clinic or lost earnings as a result of attending hospital appointments. Furthermore, we did not include additional costs associated with severe visual impairment (e.g. canes, costs of injuries and burns, etc.). Data on healthcare resource utilization in Malawi or in a similar setting is very sparse and, to the best of our knowledge, there are no studies reporting this information. However, we believe this to be a conservative approach. Additional healthcare resource utilization related to severe vision impairment would be associated with an increase in costs larger in the no intervention arm than in the treatment arm, with a consequent reduction of the incremental costs which would favor laser treatment in terms of cost-effectiveness. Our model also fails to take into account the impact of vision loss on other household members. This might include the cost of informal care provided and additional income loss, both of which could affect the patient’s welfare.

### Interpretation

Based on the results of our analysis, the introduction of a program of targeted photographic screening and laser treatment for STDR, in a public hospital setting in Malawi, appears to be cost-effective in a variety of service delivery scenarios. The most cost-effective scenarios that we identified involved treating a younger cohort than the one in the base case, and achieving a high rate of usage of the service.

In our model retinal photography, grading and laser treatment were conducted by an ophthalmologist. Depending on the setting, salary costs might be lower if some of these steps were performed by allied health professionals, rather than doctors. We have included the cost of a fundus camera in our calculations. It is a valuable tool for documenting retinal findings, monitoring progress and explaining DR to patients. Screening programs in both high and low resource settings require quality assurance procedures. Our experience in the UK suggests that photographic screening is the best method to achieve this. Technological developments which reduce equipment costs would be expected to improve the cost-effectiveness of the intervention.

### Generalizability

This project was conducted in urban and semi-urban regions of Malawi where, although the prevalence of diabetes has been shown to be similar [12], the risk factors for the development and progression of STDR might be different from those in a rural context. As a result, it remains unclear whether the cost-effectiveness of this approach to screening and treatment for STDR would be the same in a more rural environment. The results that we have shown here are likely to be most generalizable to other urban and semi-urban low-income country settings. Our screening was targeted at people with diabetes attending a routine diabetes clinic, a requirement for access to medications. We believe this model is realistic and sustainable in a resource poor setting such as Malawi where community based or primary care screening is unaffordable and impractical. If the distribution of patients amongst the health states and the clinical outcomes are similar in other low-income countries with a higher GDP than that of Malawi, then the resulting increase in the WHO threshold would favor the cost-effectiveness of the intervention in these countries.

In summary, we have shown that a targeted screening and laser treatment service for STDR in Malawi can be cost-effective, by the standards set by the WHO. Our study highlights the ongoing need for data collection on the epidemiology of DR across sub-Saharan Africa to inform policy on clinical care for those with diabetes in this setting.

## Supporting information

S1 Data MDRS patient-level data. (XLSX)

S1 Fig Cost-effectiveness acceptability curves based on DALYs. Probability that the intervention is cost-effective in a range of cost-effectiveness thresholds for the base case, + 20% on salaries scenario, -20% on salaries scenario, 50% utilization rate scenario, and age 30 scenario. DALY = Disability-adjusted life year.
(TIF)
